# Supplementary material for: Moringa Oleifera Alleviates Aβ Burden and Improves Synaptic Plasticity and Cognitive Impairments in APP/PS1 Mice
Source: Nutrients. 2022 Oct 14;14(20):4284. doi: 10.3390/nu14204284 (PMC9609596; doi:10.3390/nu14204284)
Supplement: Supplementary file 1 [file nutrients-14-04284-s001.zip › nutrients-1901048-supplementary.pdf]

Supporting information

Supporting information Figure S1: Western blot gels for Figure 2A

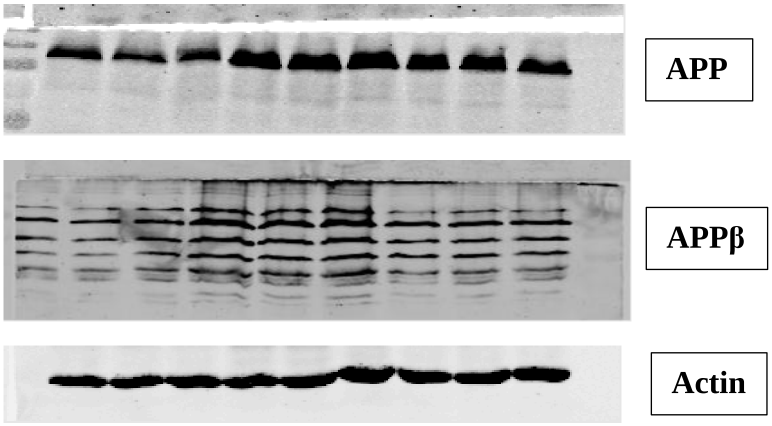

Supporting information Figure S2: Western blot gels for Figure 3A

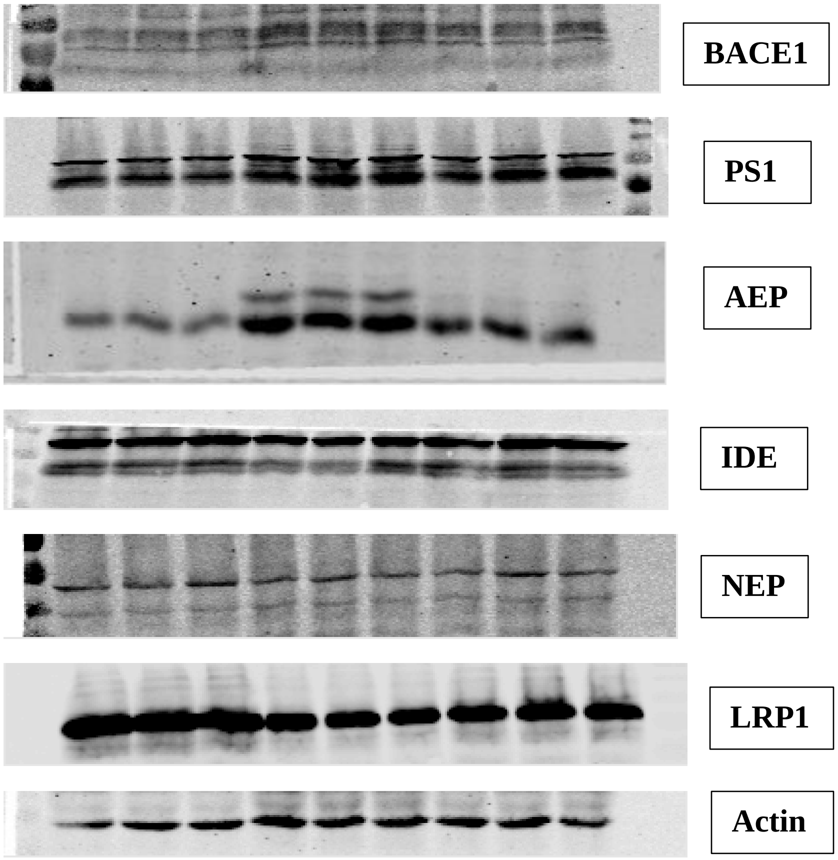

Supporting information Figure S3: Western blot gels for Figure 4A

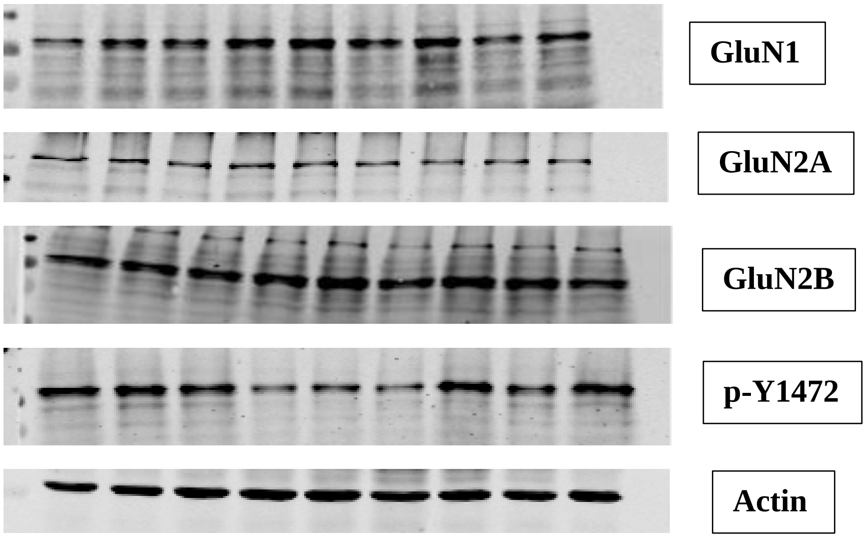

Supporting information Figure S4: Western blot gels for Figure 4F

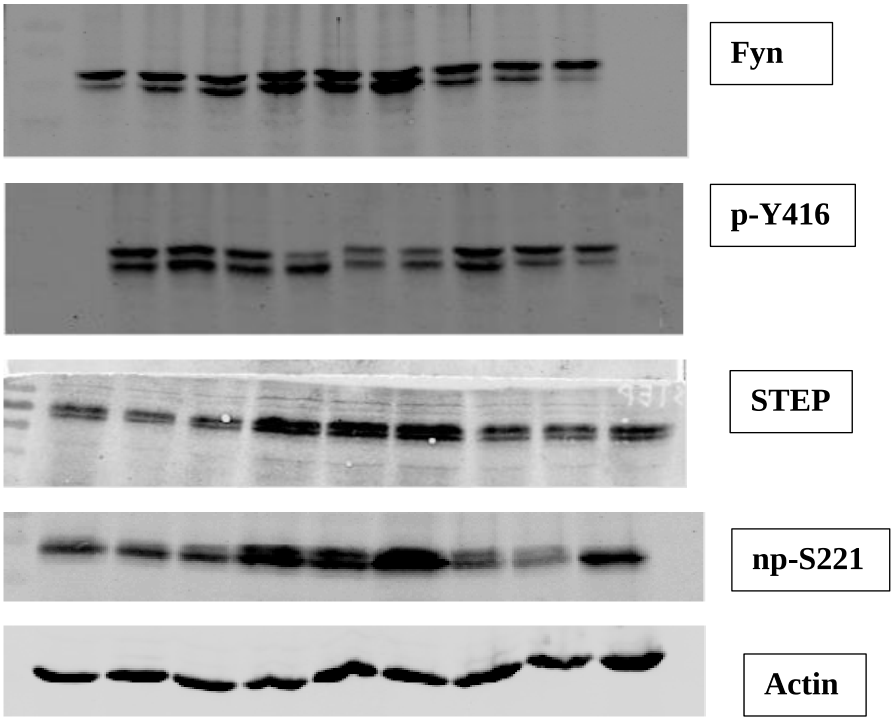

Supporting information Figure S5: Western blot gels for Figure 5A

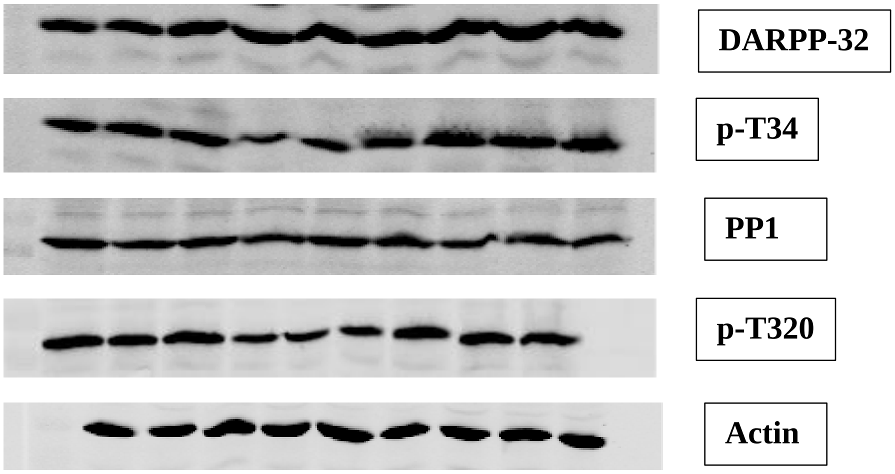

Supporting information Figure S6: Western blot gels for Figure 6A

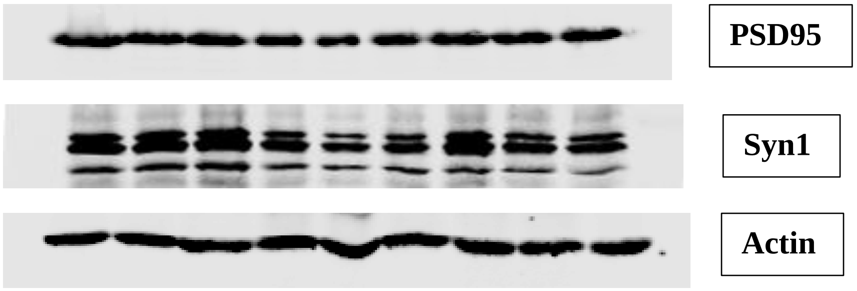

Supporting information Figure S7: Western blot gels for Figure 7A

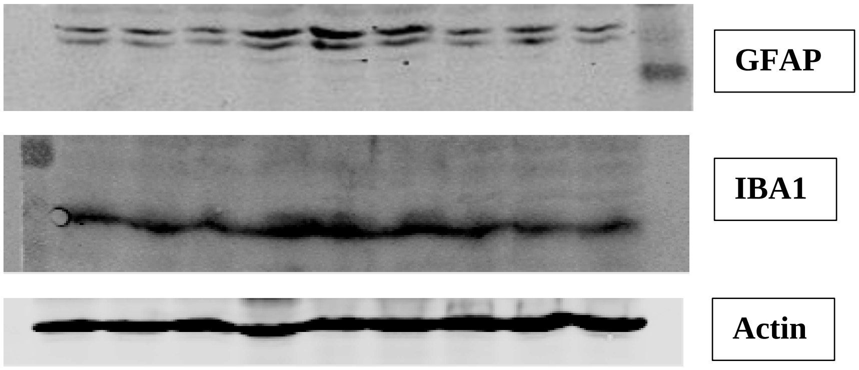

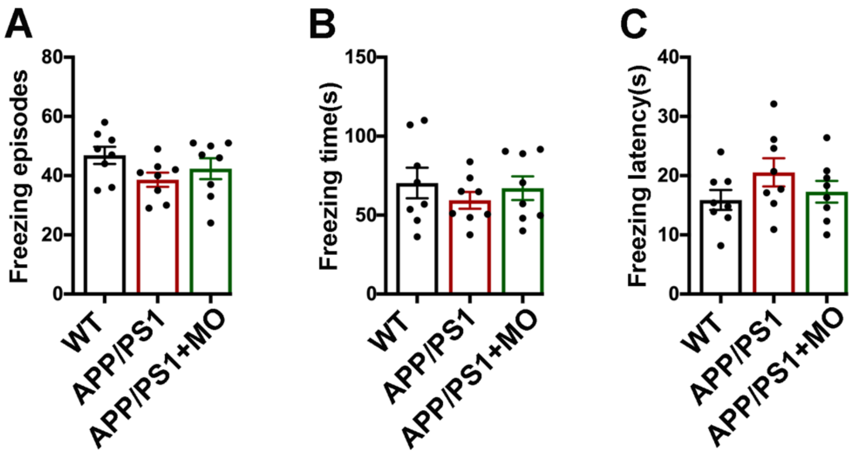

Supplementary Figure S8. No significant difference in the cued fear conditioning test results among all three groups. A. the freezing episodes; B. the freezing time (s); and C. the freezing latency (s) during the test time. Data are presented as Mean ± SEM, n=8 for each group.
